# Mucinous and Signet-Ring Cell Colonic Adenocarcinoma in Inflammatory Bowel Disease: A Case–Control Study

**DOI:** 10.3390/cancers15153803

**Published:** 2023-07-26

**Authors:** Benedetto Neri, Roberto Mancone, Luca Savino, Sara Schiavone, Vincenzo Formica, Francesca Pizzi, Silvia Salvatori, Michelangela Mossa, Stefano Migliozzi, Mariasofia Fiorillo, Cristina Morelli, Alessandro Moscardelli, Elisabetta Lolli, Emma Calabrese, Giuseppe S. Sica, Giovanni Monteleone, Livia Biancone

**Affiliations:** 1 Gastroenterology Unit, Department of Systems Medicine, University “Tor Vergata” of Rome, 00133 Roma, Italy; benedettoneri@gmail.com (B.N.); roberto.mancone@yahoo.it (R.M.); saraschiavone27@gmail.com (S.S.); francy.pizzi@gmail.com (F.P.); silviasalvatori23@gmail.com (S.S.); michelangela.mossa@gmail.com (M.M.); stefanomigliozzi946@gmail.com (S.M.); fiorillo93@gmail.com (M.F.); alessandro.moscardelli24@gmail.com (A.M.); elisabetta.lolli@ptvonline.it (E.L.); emma.calabrese@uniroma2.it (E.C.); gi.monteleone@med.uniroma2.it (G.M.); 2 Pathology Unit, Department of Biomedicine and Prevention, University “Tor Vergata” of Rome, 00133 Roma, Italy; luca.savino@ptvonline.it; 3 Medical Oncology Unit, Department of Systems Medicine, University “Tor Vergata” of Rome, 00133 Roma, Italy; v.formica1@gmail.com (V.F.); cristina.morelli89@gmail.com (C.M.); 4 Department of Surgery, University “Tor Vergata” of Rome, 00133 Roma, Italy; sigisica@gmail.com

**Keywords:** colorectal cancer, mucinous adenocarcinoma, signet-ring cell adenocarcinoma, inflammatory bowel disease

## Abstract

**Simple Summary:**

Chronic active inflammation is a known risk factor for colorectal cancer (CRC) in inflammatory bowel disease (IBD), while the adenoma–carcinoma sequence appears to be associated with sporadic CRC. In the general population, mucinous and signet-ring cell adenocarcinomas are characterized by a worse prognosis. In IBD, a higher frequency of these CRC histotypes has been reported. In the present study, we investigated the frequency and characteristics of mucinous and signet-ring cell adenocarcinomas in patients with IBD versus age-matched non-IBD Controls. CRC was more frequently represented by mucinous/signet-ring cell adenocarcinoma in IBD than in Controls. In rectal CRC, there was a higher proportion of mucinous/signet-ring cell adenocarcinoma vs. standard adenocarcinoma in IBD but not in non-IBD Controls. No risk factors for these two CRC histotypes were identified in IBD. Present findings support that the frequency of mucinous/signet-ring cell colorectal adenocarcinoma is higher in IBD, being associated with rectal involvement of CRC.

**Abstract:**

A higher frequency of mucinous and signet-ring cell colonic adenocarcinoma has been reported in inflammatory bowel disease (IBD). The primary aim was to investigate the frequency of mucinous and signet-ring cell colorectal adenocarcinoma in patients with IBD (Cases) versus age-matched non-IBD Controls. The secondary aims were to compare the characteristics of these two histotypes of colorectal cancer (CRC) in IBD patients vs. Controls and to search for specific risk factors in IBD. In a case–control study, all IBD patients with CRC diagnosed from 2000 to 2022 were enrolled and matched for age (1:2) with non-IBD Controls with CRC. The study population included 120 CRC patients (40 IBD, 80 Controls). In IBD, CRC included standard adenocarcinoma in 23 (57.5%) patients mucinous/signet-ring cell adenocarcinoma in 17 (42.5%) patients. The proportion of mucinous/signet-ring cell adenocarcinoma was higher in IBD than in Controls (17 [42.5%] vs. 18 [22.5%]; *p* = 0.03). In rectal CRC, the proportion of mucinous/signet-ring cell adenocarcinoma was higher than standard adenocarcinoma in IBD (8 [47.1%] vs. 4 [17.4%]; *p* = 0.04) but not in Controls (4 [22.2%] vs. 20 [32.2%]; *p* = 0.59). In rectal CRC, the proportion of these two histotypes was higher in Cases than in Controls (8/12 [66.6%] vs. 4/24 [16.6%]; *p* = 0.008), with no risk factors identified in IBD. CRC was more frequently represented by mucinous/signet-ring cell adenocarcinoma in IBD than in age-matched non-IBD Controls. In IBD, these two CRC histotypes were more frequent in the rectum.

## 1. Introduction

Inflammatory bowel diseases (IBDs) are chronic diseases including Crohn’s disease (CD) and ulcerative colitis (UC) [1,2]. Colorectal cancer (CRC) risk is increased in IBD, particularly in patients with colonic involvement of the lesions, thus representing one of the major complications in these patients. A lower median age at diagnosis of CRC has been reported in IBD [3]. Long-standing (>8–10 years) IBD colitis is associated with a higher risk of CRC [4]. IBD duration correlates with CRC risk, increasing from 1 to 2% at 10 years, 4 to 8% at 20 years, and 14 to 18% at 30 years after IBD diagnosis [5,6]. In IBD involving >1/3 of the colon, CRC risk is 1.4–2.2 times higher than in the general population [7]. Besides risk factors for CRC common to the general non-IBD population [4,5,6], additional risk factors in IBD include chronically active disease in the colon–rectum, concomitant primary sclerosing cholangitis (PSC), and persistent histological inflammation [6,8,9,10,11]. Treatments for IBD, including biologics, currently do not appear to increase CRC risk in these patients [7].

Endoscopic surveillance tailored according to these variables is therefore recommended in IBD [4].

While sporadic CRC is known to originate from the adenoma–carcinoma sequence, persistent active inflammation of the colon is currently identified as a key risk factor for the development of CRC in IBD [12]. Chronic inflammation may indeed determine diffuse DNA damage within the colorectal epithelial cells, thus leading to multiple foci of aberrant neoplastic cells [13]. Current evidence suggests that different pathogenetic mechanisms are involved in sporadic versus colitis-associated CRC. In IBD, CRC has been associated with chromosomic instability involving TP53 mutation [14].

In the general population, mucinous and signet-ring cell adenocarcinomas are characterized by a worse prognosis [15]. In IBD, a higher frequency mucinous and signet-ring colonic adenocarcinoma has been reported [16]. However, risk factors for these aggressive CRC histotypes have not yet been defined.

Based on these observations, the primary aim of the present case–control study was to investigate whether the frequency of mucinous and signet-ring cell adenocarcinoma is higher in patients with IBD versus age-matched non-IBD Controls. The secondary aim was to search for differences in terms of localization of these two CRC histotypes in patients with IBD versus non-IBD Controls. Risk factors for colorectal mucinous and signet-ring cell adenocarcinoma in IBD were also assessed.

## 2. Methods

### 2.1. Study Protocol

In a retrospective, 22-year case–control study, all IBD patients with a diagnosis of CRC (Cases) occurring from January 2000 to July 2022 were enrolled. For each IBD patient with CRC, 2 patients with CRC without IBD (Controls) were retrospectively enrolled. Cases and Controls were matched for age (±5 years). IBD was diagnosed and classified according to standard criteria [1,2], including the Montreal classification [17]. A histological report of the surgical sample was required for diagnosing CRC. Clinical data referred to the last available follow-up visit.

### 2.2. Study Population

Inclusion criteria for all patients were (1) age ≥ 18, (2) well-defined diagnosis of CRC, (3) available histological and surgical report, (4) and clinical assessment at the University “Tor Vergata” of Rome, at any time during the history of CRC. Additional inclusion criterium for Cases was (a) a well-defined diagnosis of IBD [1,2]. The exclusion criterium for all patients was: (1) Missing demographic or clinical data.

Demographic and clinical characteristics of Cases and Controls were reported in a database and included: age, gender, age at CRC diagnosis, CRC histotype (standard, mucinous, signet-ring cell adenocarcinoma), and localization. Additional data considered for IBD patients were CRC stage at diagnosis, surgical therapy for CRC (yes/no, type), neo-adjuvant (yes/no, type) and/or adjuvant chemo- or radiotherapy (yes/no, type), survival (yes/no), the time interval from CRC diagnosis to death (months), smoking status (yes vs. no/ex), IBD duration (years), UC extent (proctitis, E1; left-sided, E2; extensive, E3) [17], CD location (ileum, L1; colon, L2; ileum-colon, L3; upper GI, L4), CD behavior (non-fibrostricturing non-penetrating, B1; fibrostricturing, B2; penetrating, B3) [17], perianal disease (yes/no), IBD-related surgery (yes/no), extraintestinal manifestations (EIMs) (yes/no, type), prior (at any time from the diagnosis of IBD to the diagnosis of CRC) or ongoing (≤3 months from CRC diagnosis) treatment with thiopurines, methotrexate, biologics (yes/no, type), family history of CRC (yes/no), history of adenomas or dysplasia of the colon (yes/no), CRC-related symptoms (yes/no, type), and modality of diagnosis of CRC.

The study protocol was approved by the local independent Ethics Committee (CEI) of the Policlinico “Tor Vergata” of Rome, Italy (Protocol No.: 166.22). Patient consent was waived as data were retrospectively collected, the investigation did not add risk for participants, and all data were deidentified.

### 2.3. Statistical Analysis

Data were expressed as median [range]. The normal distribution of parametric continuous variables was assessed by using the Kolmogorov–Smirnov test and defined by *p* > 0.05. Differences between qualitative and quantitative variables were assessed by the Pearson χ^2^ test, Student’s *t*-test, or Mann–Whitney U-test, as appropriate. Univariate and multivariate logistic regression models were used for assessing risk factors for IBD activity (OR [95% CI]). Survival analysis was performed according to the Kaplan–Meier curves and the log-rank test. Statistical significance was considered for all variables in the case of *p* < 0.05. Statistical analysis was performed with IBM SPSS statistical software vers. 26.0.

## 3. Results

### 3.1. IBD Patients with CRC

Overall, 40 IBD patients with concomitant CRC (Cases) diagnosed from January 2000 to July 2022 were enrolled. Demographic and clinical characteristics of IBD patients with CRC are summarized in Table 1 (median, years (range): age 58 (30–84), age at diagnosis of IBD 35.5 (29–80), IBD duration 21 (1–48), age at diagnosis of CRC 54 (29–80), the time interval from IBD diagnosis to CRC 16 (1–45). IBD patients with CRC included 24 (60%) patients with UC and 16 (40%) with CD. None of the 40 IBD patients had a history of PSC.

### 3.2. Colorectal Cancer in IBD

Histological analysis showed that in the 40 IBD patients, CRC included standard adenocarcinoma in 23 (57.5%) and mucinous or signet-ring cell adenocarcinoma in 17 (42.5%) patients. Specifically, in the IBD group, there were 9 (22.5%) mucinous and 8 (20%) signet-ring cell adenocarcinomas. Characteristics of CRC in patients with IBD are summarized in Table 2. As shown, at the time of diagnosis of CRC, cancer-related symptoms were observed in 16 (40%) patients, showing rectal bleeding in 1 (6.2%), abdominal pain in 4 (25%), refractory IBD in 5 (31.3%), and obstructive symptoms in 6 (37.5%) patients. CRC was diagnosed by using colonoscopy with biopsy samples in 32 (80%) patients and by cross-sectional imaging in 3 (7.5%) patients, while intraoperative diagnosis was made in 5 (12.5%) patients. Concomitant adenomas were detected in 5 (12.5%) patients, history of adenomas or dysplasia in 10 (25%) patients, while no IBD patients had a previous history of CRC. Family history of CRC was observed in 7 (17.5%) patients. In IBD, CRC involved the rectum in 12 (30%), the sigmoid colon in 9 (22.5%), the descending colon in 8 (20%), the transverse colon in 2 (5%), and the ascending colon in 9 (22.5%) patients. CRC staging at diagnosis and medical and surgical treatment for cancer are reported in Table 2.

The median survival time after the diagnosis of CRC (from the diagnosis to the last visit/death) was 61.5 (1–269) months. In particular, the survival after incident CRC was comparable between patients with mucinous vs. signet-ring cell adenocarcinoma (22.5 (6–109) vs. 15 (4–111); *p* = 0.64). CRC-related death occurred in 8 (20%) IBD patients, 21 patients are in follow-up, while 11 (27.5%) patients were lost to follow-up.

### 3.3. Standard versus Mucinous/Signet-Ring Cell Adenocarcinoma in IBD

**Figure 1:**
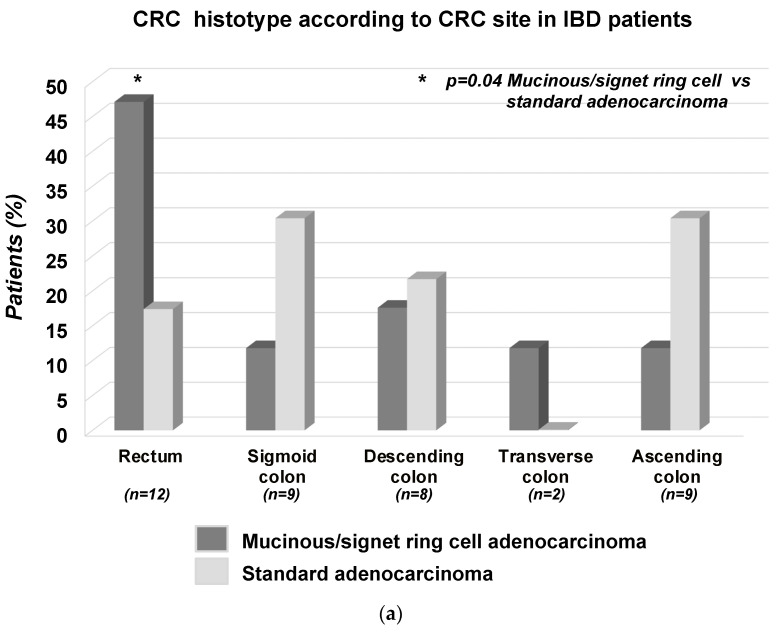

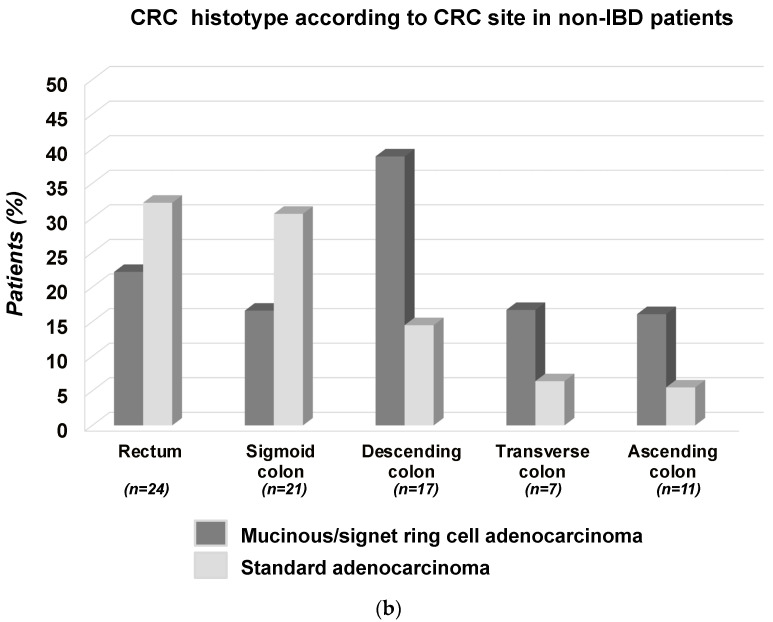
(**a**,**b**): Proportion of mucinous/signet-ring cell adenocarcinoma versus standard adenocarcinoma in different colorectal segments in patients with IBD (**a**) and, separately, non-IBD-Controls (**b**). (**a**) In IBD patients, rectal CRC was more frequently represented by mucinous/signet-ring cell than standard adenocarcinoma (*p* = 0.04). Differently, the frequency of these 2 CRC histotypes in the other colonic segments was comparable (sigmoid colon *p* = 0.31; descending colon *p* = 0.93; transverse colon *p* = 0.34; coecum/ascending colon *p* = 0.31). (**b**) Differently from IBD patients, in non-IBD Controls, the frequency of standard versus mucinous/signet-ring cell adenocarcinoma was comparable in each colorectal segment involved by CRC (rectum: *p* = 0.59; sigmoid colon: *p* = 0.25; descending colon *p* = 0.08; transverse colon *p* = 0.38, coecum/ascending colon: *p* = 0.44).

In the tested IBD population, rectal CRC was more frequently represented by mucinous/signet-ring cell adenocarcinoma than standard adenocarcinoma (8 [47.1%] vs. 4 [17.4%]; *p* = 0.04) (Figure 1a). In the same IBD population, a comparable frequency of mucinous/signet-ring cell versus standard adenocarcinoma was observed when CRC involved the other colonic segments (sigmoid colon *p* = 0.31; descending colon *p* = 0.93; transverse colon *p* = 0.34; coecum/ascending colon *p* = 0.31) (Figure 1a). Overall, among the ’0 IBD patients with CRC, cancer occurred in colonic segments involved by IBD lesions in 25 (62.5%) patients. When considering the subgroup of 35 IBD patients with colorectal involvement of IBD, CRC occurred in an involved IBD segment in 25 (71.4%) patients. In this sub-population also, the proportion of patients with mucinous/signet-ring cell vs. standard adenocarcinoma was comparable (12/14 [85.7%] vs. 13/21 [61.9%]; *p* = 0.25).

Among the 12 rectal CRCs, there were 8 mucinous/signet-ring cell and 4 standard adenocarcinomas occurring in 9 UC and 3 CD patients (1 colonic, 1 ileo-colonic, 1 ileal CD, all 3 with perianal CD). Among the 8 patients with rectal mucinous (n = 4) or signet-ring cell adenocarcinoma (n = 4), there were 5 UC and 3 CD patients (1 colonic, 1 ileo-colonic, 1 ileal CD, all 3 with perianal CD). Differently, all four IBD patients with rectal standard adenocarcinoma had UC. In patients with rectal CRC, IBD type was equally distributed among patients with standard vs. mucinous/signet-ring cell adenocarcinoma (UC: 4/4 vs. 5/8, respectively; *p* = 0.47). Demographic and clinical characteristics were comparable between IBD patients with mucinous/signet-ring cell versus standard adenocarcinoma, including CRC stages at diagnosis of cancer (Table 1).

At univariate analysis, none of the tested risk factors was significantly associated with the occurrence of mucinous/signet-ring cell adenocarcinoma in IBD (Table 3).

### 3.4. Non-IBD Patients with CRC

Overall, 80 patients with CRC without a diagnosis of IBD, matched for age (±5 years) with 40 IBD patients with CRC, were enrolled. In this control group, CRC was defined as mucinous/signet-ring cell adenocarcinoma in 18 (22.5%) and as standard adenocarcinoma in 62 (77.5%) patients. In non-IBD Controls, demographics and clinical characteristics did not differ between patients with either mucinous/signet-ring cell or standard adenocarcinoma (female gender: 12 [66.6%] vs. 3 [37%] vs. *p* = 0.05; median age at diagnosis of CRC 54 (32–83) vs. 58 (31–81) years; *p* = 0.4).

Differently from IBD patients, in the tested non-IBD population, a comparable frequency of standard versus mucinous/signet-ring cell adenocarcinoma was observed in each colorectal segment involved by CRC (rectum: 20 [32.2%] vs. 4 [22.2%]; *p* = 0.59, sigmoid colon: 19 [30.6%] vs. 3 [16.6%]; *p* = 0.25, descending colon: 10 [16.1%] vs. 7 [38.9%]; *p* = 0.08, transverse colon: 4 [6.4%] vs. 3 [16.7%]; *p* = 0.38, coecum/ascending colon: 10 [16.1%] vs. 1 [5.5%]; *p* = 0.44) (Figure 1b).

### 3.5. Characteristics of CRC in Patients with versus without IBD

When comparing patients with CRC with or without IBD, no significant differences were observed in terms of gender distribution (females: 13 [32.5%] vs. 35 [43.7%]; *p* = 0.32) and median age at diagnosis of CRC (54 (29–80) vs. 58 (31–83); *p* = 0.19).

**Figure 2:**
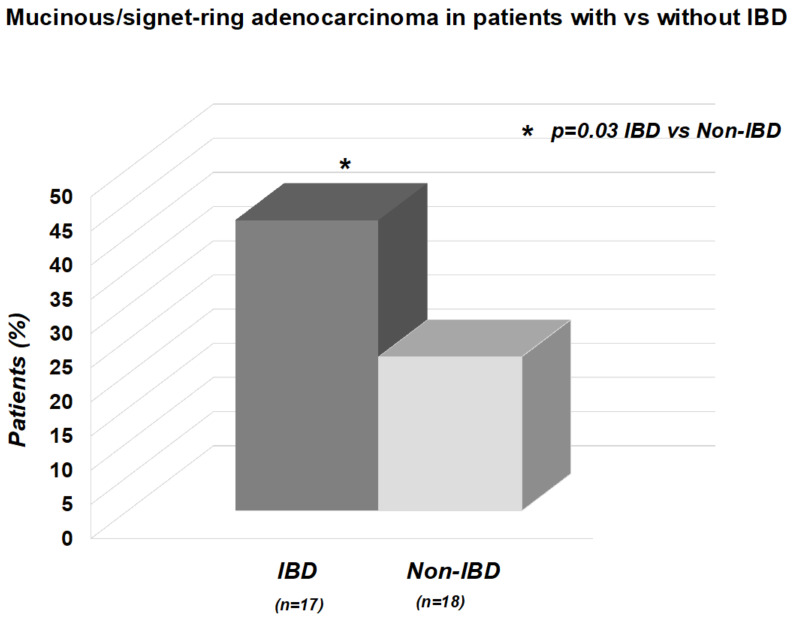
The frequency of mucinous/signet-ring cell adenocarcinoma was significantly higher in patients with IBD than in the tested age-matched non-IBD Controls (*p* = 0.03).

Histological analysis of CRC detected a significantly higher frequency of mucinous/signet-ring cell adenocarcinoma in patients with IBD than in age-matched non-IBD Controls (17 [42.5%] vs. 18 [22.5%]; *p* = 0.03) (Figure 2). The overall frequency of CRC in each colorectal segment did not differ between Cases and Controls (rectum: 12 [30%] vs. 24 [30%], *p* = 0.83; sigmoid colon: 9 [22.5%] vs. 21 [26%], *p* = 0.82; descending colon: 8 [20%] vs. 17 [21.3%], *p* = 0.93; transverse colon: 2 [5%] vs. 7 [8%], *p* = 0.71; coecum/ascending colon: 9 [22.5%] vs. 11 [13.7%], *p* = 0.34).

**Figure 3:**
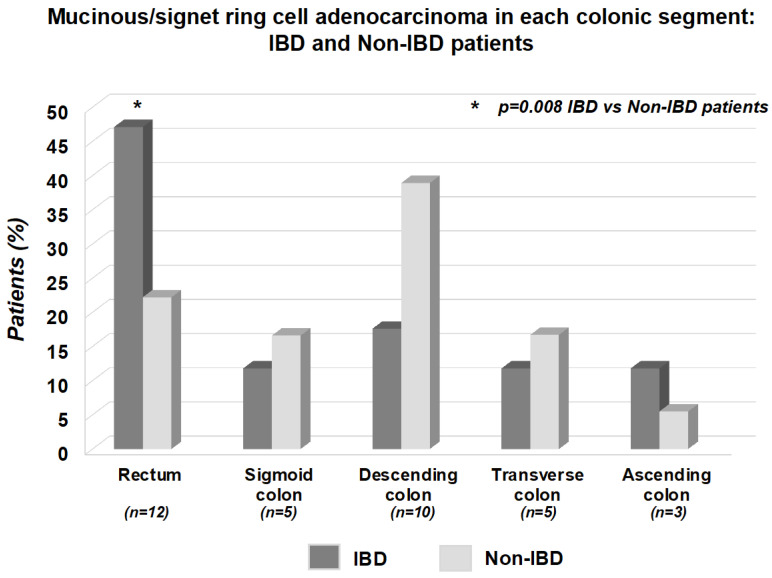
Proportion of mucinous/signet-ring cell adenocarcinoma in patients with IBD versus non-IBD Controls when separately considering each colorectal segment involved by CRC. As shown, in CRC involving the rectum, the proportion of mucinous/signet-ring cell adenocarcinoma was higher in IBD than in non-IBD Controls (*p* = 0.008). Conversely, the distribution of these 2 CRC histotypes was comparable between Cases and Controls when considering the other colorectal segments (sigmoid colon: *p* = 0.99; descending colon: *p* = 0.79; transverse colon: *p* = 0.53; coecum/ascending colon: *p* = 0.85).

In CRC involving the rectum, the proportion of mucinous/signet-ring cell adenocarcinoma was higher in IBD than in non-IBD Controls (8/12 [66.6%] vs. 4/24 [16.6%], *p* = 0.008) (Figure 3). Conversely, the distribution of these 2 CRC histotypes did not significantly differ between Cases and Controls when considering the other colorectal segments (sigmoid colon: 2/9 [22.2%] vs. 3/21 [14.3%]; *p* = 0.99, descending colon: 3/8 [37.5%] vs. 7/17 [41.2%]; *p* = 0.79, transverse colon: 2/2 [100%] vs. 3/7 [42.8%]; *p* = 0.53, coecum/ascending colon: 2/9 [22.2%] vs. 1/11 [9.1%]; *p* = 0.85) (Figure 3).

## 4. Discussion

CRC is one of the most feared complications of IBD. CRC in IBD is currently characterized by a diagnosis occurring at younger age and at advanced stages when compared to the general non-IBD population [3]. Thus, tremendous efforts have been made to develop tailored screening programs aimed to allow a timely diagnosis and treatment for CRC in IBD [4]. Recently, technological improvements, including chromoendoscopy rather than random biopsy sampling, improved the search for colonic dysplasia [4,18]. Higher diagnostic rates are therefore expected to reduce the diagnostic delay, thus allowing an earlier diagnosis of CRC [4]. Nevertheless, CRC still represents a major concern and one of the causes of mortality in IBD, even in patients at a young age. Besides recognition of dysplasia, identifying additional risk factors for CRC in patients with IBD is also required for optimizing the prevention, early diagnosis, and treatment of cancer. Several IBD-related risk factors for CRC have been identified so far [6,8,9,10,11]. Active microscopic inflammation rather than post-inflammatory polyps has recently been reported as a risk factor for colorectal neoplasia in patients with IBD [19,20].

CRC in IBD is more frequently represented by standard adenocarcinoma, as observed in the general non-IBD population [21]. However, a higher frequency of mucinous and signet-ring cell adenocarcinoma has been suggested in IBD [16,22]. In the general population, these CRC histotypes, particularly signet-ring cell adenocarcinoma, are associated with a worse outcome when compared to standard adenocarcinoma. Whether this is true also for patients with IBD is undefined, as also potential associated risk factors.

In our IBD population, the frequency of mucinous/signet-ring cell colorectal adenocarcinoma was higher than in non-IBD Controls. This observation is in agreement with previous findings [16,22]. Different pathogenetic mechanisms leading to CRC have been hypothesized in IBD [23]. Non-conventional dysplasia has been associated with low-grade tubuloglandular and mucinous adenocarcinomas in IBD [24]. Molecular alterations, including a high frequency of c-MYC amplification associated with mucinous and signet-ring cell differentiation, have been reported in IBD-associated intestinal adenocarcinomas [25]. In CRC occurring in IBD, P53 alteration and microsatellite instability due to long-standing chronic inflammation appears to be involved in carcinogenesis [25]. Similarly, microsatellite instability is a molecular pathway suggested to be associated with mucinous adenocarcinoma [26]. The observed differences between our tested IBD versus non-IBD population in terms of frequency and distribution of mucinous/signet-ring cell colorectal adenocarcinoma add support to the need for further studies in this regard.

In our IBD study population, the frequency of mucinous/signet-ring cell adenocarcinoma localized in colorectal segments involved by IBD was high (70.6%), although comparable to the frequency observed in patients with standard adenocarcinoma (56.5%). Recently, a multicenter study reported the possible association between neuroendocrine neoplasms and inflamed segments in IBD [27]. The same was not observed in the present study, which focused on colorectal adenocarcinoma. Moreover, discrepancies between endoscopic and microscopic activity may occur in IBD [28]. The possible relationship between microscopic lesions related to IBD and the development of specific CRC histotypes is currently undefined.

Of interest, in the tested IBD population, CRC localized in the rectum was more frequently represented by mucinous/signet-ring cell than standard adenocarcinoma. The same was not observed in the age-matched non-IBD population. Moreover, mucinous/signet-ring cell adenocarcinoma involving the rectum, but not the other colonic segments, was more frequent in IBD than in non-IBD Controls. In the general non-IBD population, a higher proportion of mucinous adenocarcinoma has been reported in the proximal colon, although other studies showed a higher frequency in the rectum [29]. Although limitations related to the few observed cases, in our series of IBD patients with rectal mucinous/signet-ring cell adenocarcinoma, seven out of eight (87.5%) patients had rectal involvement of the disease. This observation may be related to IBD-related chronic colitis, which currently appears as the main pathogenetic mechanism in these patients. The sequence chronic inflammation-dysplasia-cancer, described in IBD-related CRC, has been reported to determine cellular mutations different from those observed in sporadic CRC. The involvement of reactive oxygen and nitrogen species produced by chronic inflammation, alterations in microbiota, and a dysregulation of the host immune response have been suggested in the development of neoplasia in IBD [30]. In a French study, a high risk of anal and rectal cancer has been reported in patients with anal and/or perianal CD [31]. In our study, perianal disease was observed in three out of eight CD patients with rectal cancer, thus not allowing conclusive statements in this regard.

Data regarding gender distribution and the median age at diagnosis of CRC between mucinous/signet-ring cell versus standard colorectal adenocarcinoma in non-IBD patients are conflicting [29,32,33]. In our IBD and non-IBD populations, these characteristics were comparable between patients with these two histotypes of CRC, in agreement with some of the studies in this regard [29,32].

In the tested IBD population, an overall low proportion of CRC-related symptoms (40%) has been observed, being mostly represented by refractory IBD. This further supports the need for tailored surveillance programs aimed to prevent or allow a timely diagnosis of CRC, most often mimicking IBD-related symptoms. Accordingly, our previous multicenter study reported an overall low frequency of symptomatic CRC in IBD, thus also highlighting the issue of appropriate CRC surveillance in tertiary IBD referral centers [34].

In our IBD population, patients without colorectal inflammation were not excluded in order to search for potential additional risk factors for CRC not related to chronic inflammation. Moreover, CD localization (including ileal disease in only 5 patients) did not represent a significant risk factor for CRC at univariate analysis (*p* = 0.4). Overall, no risk factors for mucinous/signet-ring cell adenocarcinoma were identified. To our knowledge, no defined risk factors for these histotypes have been identified so far, and female gender has been suggested, with conflicting findings [29,32,33]. In the tested IBD population, gender distribution was not identified as a risk factor for mucinous/signet-ring cell adenocarcinoma.

A worse prognosis has been reported in patients with mucinous/signet-ring cell vs. standard colorectal adenocarcinoma [35], although with discrepant findings [29,36]. In our IBD population, survival was comparable in patients with the two CRC histotypes. Leopoldo et al. [29] hypothesized that mucinous/signet-ring cell adenocarcinoma of the colon–rectum does not carry a worse outcome per se, but rather in relation to molecular alteration associated with cancer subtype, showing different features, outcomes, and drug responsiveness. However, the high frequency (27.5%) of IBD patients lost to follow-up and the missing data regarding the non-IBD control population, may have a role in this finding. Moreover, outcome analysis and mortality evaluation did not represent the primary or secondary aims of this study.

Among the limitations of the present study, there is the retrospective study design and, therefore, the lack of a sample size calculation. Moreover, as our non-IBD control population was recruited from the Oncology Unit, all Controls had advanced CRC, thus not allowing comparisons with IBD patients in terms of survival after CRC. Strengths of the study include characteristics of the tested IBD patients, represented by a homogeneous population comparable to the general IBD population [1,2], thus reducing the risk of selection bias. The tested IBD population considered was quite small. Nevertheless, to our knowledge, limited data are currently available regarding characteristics and risk factors for mucinous/signet-ring cell adenocarcinoma in patients with IBD.

## 5. Conclusions

Findings from the present study add support to the concept that the frequency of mucinous/signet-ring cell colorectal adenocarcinoma is higher in IBD. Present observations also suggest that mucinous/signet-ring cell colorectal adenocarcinoma may be associated with characteristics of IBD, specifically with rectal involvement of CRC. The evaluation of molecular alterations of CRC and particularly of mucinous/signet-ring cell adenocarcinoma in IBD may enlighten about mechanisms involved in the development of this aggressive cancer in these patients.

## Figures and Tables

**Table 1:** Demographic and clinical characteristics of IBD patients with colorectal adenocarcinoma.

	IBD Patients (n = 40)	Standard Adenocarcinoma (n = 23)	Mucinous/Signet-Ring Cell Adenocarcinoma (n = 17)	*p*
Age at diagnosis of CRC, median (range)	54 (29–80)	61 (30–80)	53 (29–80)	0.61
Age at diagnosis of IBD, median (range)	35.5 (29–80)	38 (12–80)	29 (17–73)	0.56
Gender (F), n (%)	13 (32.5%)	8 (34.8%)	5 (29.4%)	0.98
IBD duration, median (range)	21 (1–48)	22 (3–48)	21 (1–38)	0.77
Time interval between diagnosis of IBD and CRC, median (range)	16 (1–45)	14 (1–45)	17 (1–36)	0.74
Ulcerative colitis, n (%)	24 (60%)	14 (60.9%)	10 (58.8%)	0.84
E1	7 (29.2%)	4 (28.6%)	3 (30%)	0.70
E2	5 (20.8%)	3 (21.4%)	2 (20%)	0.67
E3	12 (50%)	7 (50%)	5 (50%)	0.67
Crohn’s disease, n (%)	16 (40%)	9 (39.1%)	7 (41.2%)	0.84
L1	5 (31.3%)	2 (22.2%)	3 (42.8%)	0.73
L2	3 (18.7%)	1 (11.1%)	2 (28.6%)	0.8
L3	8 (50%)	6 (66.7%)	2 (28.6%)	0.31
L4	0 (0%)	0 (0%)	0 (0%)	N/A
B1	4 (25%)	1 (11.1%)	3 (42.8%)	0.38
B2	8 (50%)	7 (77.8%)	1 (14.4%)	0.04
B3	4 (25%)	1 (11.1%)	3 (42.8%)	0.38
Perianal disease, n (%)	8 (50%)	3 (33.3%)	5 (29.4%)	0.37
IBD-related surgery, n (%)	12 (30%)	7 (77.8%)	5 (29.4%)	0.78
Smoking habits, n (%)				
Yes	3 (7.5%)	1 (4.3%)	2 (11.7%)	0.78
No/Ex	37 (92.5%)	22 (95.7%)	15 (88.3%)	
EIMs, n (%)	7 (17.5%)	4 (44.4%)	3 (17.6%)	0.68
Thiopurines, n (%)	11 (27.5%)	5 (21.7%)	6 (35.3%)	0.55
Biologics, n (%) *	11 (27.5%)	6 (26.1%)	5 (29.4%)	0.9
Infliximab	9	5	4	
Adalimumab	5	1	4	
Golimumab	1	1	0	
Vedolizumab	2	2	0	
Ustekinumab	1	1	0	

* 1 UC patient required 3 biologics, 4 IBD patients 2 biologics (1 UC, 3 CD). Abbreviations: IBD = inflammatory bowel disease; CRC: colorectal cancer; F: female; E1: proctitis; E2: left-sided colitis; E3: pancolitis; L1: ileum; L2: colon; L3: ileum–colon; L4: upper gastro-intestinal tract; B1: non-fibrostricturing non-penetrating; B2: fibrostricturing; B3: penetrating; EIMs: extraintestinal manifestations; N/A: not applicable.

**Table 2:** Standard vs. mucinous/signet-ring cell colorectal adenocarcinoma in patients with IBD.

	IBD Patients (n = 40)	Standard Adenocarcinoma (n = 23)	Mucinous/Signet-Ring Cell Adenocarcinoma (n = 17)	*p*
CRC symptoms, n (%)	16 (40%)	8 (34.8%)	8 (47.1%)	0.64
CRC diagnostic modality				
Colonoscopy	32 (80%)	20 (86.9%)	12 (70.6%)	0.37
Imaging	3 (7.5%)	0 (0%)	3 (17.6%)	0.13
Intraoperative	5 (12.5%)	3 (13.1%)	2 (11.8%)	0.71
Concomitant adenoma, n (%)	5 (12.5%)	4 (17.4%)	1 (5.9%)	0.54
Previous history of adenoma, n (%)	10 (25%)	7 (30.4%)	3 (17.6%)	0.57
Family history of CRC, n (%)	7 (12.5%)	5 (21.7%)	2 (11.8%)	0.68
CRC stage at diagnosis				
I	7 (17.5%)	6 (26.1%)	1 (5.9%)	0.21
II	13 (32.5%)	9 (39.1%)	4 (23.5%)	0.48
III	12 (30%)	5 (21.7%)	7 (41.2%)	0.32
IV	8 (20%)	3 (13.1%)	5 (29.4%)	0.37
Metastasis at CRC diagnosis, n (%)	14 (35%)	6 (26.1%)	8 (47.1%)	0.29
CRC site, n (%)				
Rectum	12 (30%)	4 (17.4%)	8 (47.1%)	0.04
Sigmoid colon	9 (22.5%)	7 (30.4%)	2 (11.8%)	0.31
Descending colon	8 (20%)	5 (21.7%)	3 (17.6%)	0.93
Transverse colon	2 (5%)	0 (0%)	2 (11.8%)	0.34
Coecum/Ascending colon	9 (22.5%)	7 (30.4%)	2 (11.8%)	0.31
Left colon	29 (72.5%)	16 (69.6%)	13 (76.5%)	0.09
Right colon	11 (27.5%)	7 (30.4%)	4 (23.5%)	
Surgery for CRC, n (%)	37 (92.5%)	20 (86.9%)	17 (100%)	0.34
Type of surgery, n (%)				
EMR	3 (7.5%)	3 (13.1%)	0 (0%)	0.34
Hemicolectomy	8 (20%)	5 (21.7%)	3 (17.6%)	0.93
Subtotal colectomy with IRA	9 (22.5%)	5 (21.7%)	4 (23.5%)	0.8
Proctocolectomy and ileostomy	10 (25%)	5 (21.7%)	4 (23.5%)	0.8
Proctocolectomy and IPAA	5 (12.5%)	2 (8.7%)	3 (17.6%)	0.71
Palliative stoma	2 (5%)	0 (0%)	2 (11.8%)	0.34
Anterior rectal resection	2 (5%)	1 (4.3%)	1 (5.9%)	0.6
Neoadjuvant RT, n (%)	1 (2.5%)	0 (0%)	1 (5.9%)	0.87
Neoadjuvant CHT, n (%)	1 (2.5%)	1 (4.3%)	0 (0%)	0.87
Adjuvant RT, n (%)	1 (2.5%)	0 (0%)	1 (5.9%)	0.87
Adjuvant CHT, n (%)	15 (37.5%)	9 (39.1%)	6 (35.3%)	0.93
CRC-related death, n (%)	8 (20%)	3 (13.1%)	5 (29.4%)	0.37
Survival time, median (range)	61.5 (1–269)	37.5 (1–269)	18 (4–111)	0.09
Lost to follow-up, n (%)	11 (27.5%)	6 (26.1%)	5 (29.4%)	0.9

Abbreviations: IBD: inflammatory bowel disease; CRC: colorectal cancer; EMR: endoscopic mucosal resection; IRA: ileo-rectal anastomosis; IPAA: ileo-pouch-anal anastomosis; RT: radiotherapy; CHT: chemotherapy.

**Table 3:** Risk factors for mucinous/signet-ring cell colorectal adenocarcinoma in patients with IBD.

	Univariate Analysis	
Risk Factors	OR [95% CI]	*p*
Female gender	0.78 [0.2–3.01]	0.72
Crohn’s disease	1.08 [0.3–3.91]	0.89
Ulcerative colitis	0.91 [0.25–3.29]	0.89
IBD duration >10 years at diagnosis of CRC	2.5 [0.6–10.04]	0.19
Age at diagnosis of IBD (<40 years)	1.6 [0.46–6.09]	0.43
Smoking (yes/no)	2.93 [0.24–35.32]	0.39
Perianal disease	2.77 [0.56–13.76]	0.21
Family history of CRC	0.48 [0.08–2.83]	0.41
History of thiopurines use	1.9 [0.48–7.99]	0.88
History of biologics use	1.18 [0.29–4.77]	0.81
History of mesalazine use	0.7 [0.12–3.98]	0.68
Proctitis	1.01 [0.19–5.29]	0.98
Left-sided colitis	0.88 [0.13–6]	0.9
Pancolitis	0.95 [0.24–3.78]	0.94
Non-fibrostricturing non-penetrating CD	4.71 [0.44–49.94]	0.19
Fibrostricturing CD	0.14 [0.01–1.29]	0.08
Penetrating CD	4.71 [0.44–49.94]	0.19
Ileal CD	2.25 [0.33–15.23]	0.4
Colonic CD	2.93 [0.24–35.32]	0.39
Ileo-colonic CD	0.37 [0.06–2.16]	0.27

Abbreviations: IBD: inflammatory bowel disease; OR: odds ratio; CI: confidence interval; CRC: colorectal cancer; CD: Crohn’s disease.

## Data Availability

Data available upon request.
